# Independent, ongoing clade-specific expansions of IS5 elements in Pseudomonas syringae

**DOI:** 10.1099/mgen.0.001613

**Published:** 2026-01-30

**Authors:** David A. Baltrus, Audrey Sweten, Thomas Conomos, Nathaniel Ponvert, Jesse D. Woodson, Zachary Konkel, Jonathan Jacobs

**Affiliations:** 1 School of Plant Sciences, University of Arizona, Tucson, AZ, USA; 2 School of Animal and Comparative Biomedical Sciences, University of Arizona, Tucson, AZ, USA; 3 Department of Plant Pathology, The Ohio State University, Columbus, OH, USA

**Keywords:** *aesculi*, insertion sequence (IS) element, IS5, *lachrymans*, *Pseudomonas syringae*

## Abstract

Insertion sequence (IS) elements are transposable regions of DNA present in a majority of bacterial genomes. It is hypothesized that differences in distributions of IS elements across bacterial strains and species reflect underlying differences in population biology. Therefore, shifts in IS element distributions between closely related strains may be proxies for and reflective of changes in population dynamics. Here, we investigate the presence and distribution of a subclass of IS*5* elements throughout genomes of *Pseudomonas syringae* by querying complete genomes for the presence of InsH (the main transposase found within these IS*5* elements). We report that this one subclass of IS*5* elements appears to have recently undergone independent expansions in multiple *P. syringae* clades and find that a majority of IS*5* insertion sites are not conserved across three closely related *P. syringae* pv. *lachrymans* genomes. We present further evidence, as has been shown for other members of the IS*5* family in different taxa, that elements from this IS*5* subclass can drive the expression of downstream genes in *P. syringae*. Taken together, our results highlight how dynamic IS*5* elements can be within and across *P. syringae* genomes and point towards the potential for IS*5* elements to rewire expression patterns across the *P. syringae* genome.

**Impact Statement**

The advent of cost-efficient sequencing of complete bacterial genomes, and with it the ability to accurately sequence highly repetitive genomic regions, has highlighted the impact IS elements can have on microbial genetics and genomics. However, even with complete genomes, it can be difficult to compare patterns of IS element insertions and amplifications within a coherent phylogenetic framework to better understand evolutionary dynamics of these regions. Here, we characterize the potential for IS*5* elements to undergo rapid, ongoing expansions to >100 copies across very closely related genomes of the phytopathogen *Pseudomonas syringae*. We further demonstrate the potential of these elements to upregulate gene expression of downstream regions. Overall, our results highlight the potential for rapid expansion of IS elements to shape both coding capacity and expression potential across closely related bacterial genomes and provide a framework for analysing distributions of repetitive elements across complete genomes for all types of bacteria.

## Data Summary

Sequencing reads used for this assembly have been deposited in the SRA at accession SRR30528424. The genomic assembly of strain DBL328, generated from these reads, has been deposited at Figshare [1]. Other supplementary files and data are also available at this Figshare link.

## Introduction

Insertion sequence (IS) elements are a diverse class of transposable gene regions found throughout bacterial genomes, but are largely thought of as genomic parasites that proliferate within a genome [2,5]. Although IS elements are widespread throughout bacterial genomes and their importance in the creation of genetic and genomic novelty is clear, there have been relatively few investigations of their proliferation and evolutionary dynamics within and between lineages and species. Multiple factors contribute to this relative lack of studies; for instance, repetitive sequences the length of IS elements are often among the most fragmented regions when draft genomes are assembled from ‘short’ read sequences alone and thus it can be difficult to generate accurate counts and place IS elements in proper genomic context. Moreover, even when genomes are completely assembled, it can be challenging to compare syntenic relationships of IS elements across closely related strains. With these ideas in mind and with an interest in highlighting the evolutionary importance of IS elements, we present an analysis of the evolutionary plasticity of an IS*5* element across complete genomes of relatively closely related strains of the plant-associated and sometimes pathogenic *Pseudomonas syringae* and demonstrate the capability of this element to drive downstream gene expression in this bacterium.

IS elements are, at a minimum, composed of regions of DNA bracketed on both sides by inverted repeat sequences recognized by a transposase enzyme enabling excision/insertion of the region from/into a position in the genome [5]. In many cases, the IS element itself encodes a transposase enzyme that can recognize and excise the inverted repeat regions, enabling the entire gene to autonomously ‘jump’ or transpose to other regions of DNA within the same cell. Depending on the type of element, this jump can occur by a zero-sum ‘cut and paste mechanism’ or can be replicative and allow for proliferation of this IS element to higher copy numbers throughout the genome. Notably, multiple IS elements can combine to form compound transposons, which can carry and transfer additional beneficial genes such as those involved in antibiotic resistance [6]. Despite numerous examples where IS element disruption is highly beneficial, the overwhelming majority of evidence suggests that IS element transpositions are predominantly either neutral or deleterious [5,7,11]. Proliferation of IS elements within and across genomes is therefore thought to be governed by a combination of selection against transposition jumps that are deleterious, coupled with genetic drift (potentially through population bottlenecks) enabling proliferation and expansion of multiple copies of these genes [12], with occasional but rare selection for strong beneficial effects. For instance, in extreme cases with populations of parasitic bacteria that are bottlenecked frequently during host transmission, the number of IS elements within a genome can explode because of excess genetic drift coupled with reduced selection pressures [13]. Due to their perceived neutrality, changes in distributions of IS elements across genomes are therefore hypothesized to correlate with and potentially indicate changes in the population biology of lineages of interest.

Lastly, elements classified in the IS*5* family are a particularly interesting group because they have been relatively well-studied for their ability to ‘hotwire’ expression of genes and operons within their proximity [14,17]. Although there have been numerous examples of transcriptional upregulation due to IS*5* elements, the specific molecular events and genomic contexts enabling them to act as promoters remain unclear [14,16, 18].

*P. syringae* (*sensu lato*) is a bacterial species complex largely considered to be facultative phytopathogens commonly found associated with plants, but whose environmental persistence and transmission may also be closely tied to the water cycle [19,20]. There have now been thousands of genomes sequenced for strains designated within the *P. syringae* species complex*.* IS elements are often found within these genomes and often disrupt genes that can contribute to virulence. However, given that repetitive regions like IS elements are also the most poorly assembled parts of the genome, there have been few large-scale assessments or general comparisons of IS element compositions across *P. syringae*. Here, we present an in-depth comparison of one subclass of IS*5* elements across complete (or nearly complete) genomes, and we show that copy numbers for this particular element appear to have substantially increased independently in strains of pathovar (pv.) *aesculi* (a pathogen of horse chestnut) and pv. *lachrymans* (pathogens of cucumber). While these patterns are somewhat reflective of general IS element proliferation throughout these genomes, there is no clear general signal for proliferation across all genomes. We present further evidence comparing IS*5* element positioning across three complete pv. *lachrymans* genomes, highlighting recent and ongoing changes in the number and position of IS*5* elements. Since IS*5* elements have the potential to act as promoters enabling transcription of downstream regions in other species, we further demonstrate the ability of this element to drive gene expression of an otherwise silent antibiotic resistance gene in one of these pv. *lachrymans* strains. Taken together, our results suggest that multiple lineages of *P. syringae* have experienced extensive proliferation of an IS*5* element, and we suggest that this genomic signature could reflect changes in population biology for this lineage compared to other *P. syringae* clades. However, it is also possible that gene expression changes and disruptions caused by this element are uniquely beneficial in the context of these pv. *lachrymans* and pv. *aesculi* pathogens.

## Methods

### IS*5* element characterization across complete genomes

The IS*5* element of *P. syringae* pv. *lachrymans* was originally identified from the PGAP annotation [21] of the *Pla*107 genome. The complete protein sequence of the transposase (InsH) of this IS*5* element was used as a search query in using the DiamondblastP function against all complete genomes at https://www.pseudomonas.com/ (>1,000 at the time of query) with retention of hits that were >70% length of the original IS element and >98% protein identity [22]. All other comparative genomic data (specifically blastP hits) were acquired by accessing the DiamondblastP option at Pseudomonas.com (database version 22.1, accessed on 24 November 2023). We note that DiamondblastP will only identify IS elements with annotated InsH sequences and therefore that overall numbers represented for each strain represent a minimum number of IS*5* elements within each genome. Query sequences and a spreadsheet file of the blastP results can be found at Figshare [1].

### Characterization of all IS elements across *P. syringae* genomes

To further investigate IS element composition across *P. syringae* genomes, we queried all IS element classes within complete genome sequences which contained sequences for the InsH protein from blast searches as described above, as well as additional representative genomes (from phylogenetically informative strains). IS elements were predicted using *Pseudomonas* genome assemblies via ISEscan v1.7.2.3 with default parameters [23]. GenBank accessions for each genome queried can be found as an additional file at Figshare [1].

### Phylogenetic comparisons

We inferred phylogenetic relationships across all complete *P. syringae* genomes that contained an IS*5* element, as well as representative complete genomes from phylogenetically informative or important clades, using Realphy [24] and by designating *Pph*1448a, *Psy*B728a, *Pto*DC3000, *Por*1_4, *Pma*ES426 as references and with default parameters. Genome accessions for each of these assemblies and Realphy outputs can be found at Figshare [1].

### Comparison of IS*5* synteny and position across three complete *P. syringae* pv. *lachrymans* genomes

For comparison across three strains of *P. syringae* pv. *lachrymans*, completely sequenced chromosomes for *P. syringae* pv. *lachrymans* M301350, *P. syringae* pv. *lachrymans* NM002 and *P. syringae* pv. *lachrymans* YM7902 were queried for positions of annotated InsH sequences as above using https://www.pseudomonas.com/. We note that all three genome sequences were annotated using the NCBI PGAP pipeline, and thus annotations should be adequately comparable across all three strains. blastP was further used to search annotated protein sequences across all three genomes using loci upstream and downstream of each identified IS*5* element sequence to identify proximate regions of synteny across the three chromosomes of these strains. If a blast search failed to retrieve hits >98% for proteins of interest (or if the query was itself an IS*5* element), we queried at least three additional loci upstream or downstream of the focal IS*5* element to establish synteny. If syntenic insertion of an IS*5* element could be clearly established by hand annotation across two or three genomes or if it was obvious that the focal copy of an IS*5* element was not present within a genome, we placed this IS*5* element into a ‘clear’ group. If synteny could not easily be established due to rearrangements or other genomic variation, and the surrounding genomic region was likely present within each genome in some context, we place the focal element into an ‘unclear’ annotation group. Since we performed this search by hand, we note that there were a small number of cases where there were full-length IS*5* elements and pseudogenized IS*5* elements at syntenic positions across the genomes and we counted these as syntenic positions.

### Selection of an IS*5* element driving kanamycin gene expression

*Pseudomonas amgydali* pv. *lachrymans* 107 (*Pla*107, also known as MAFF301315 and *Pla*N7512) was originally isolated from diseased cucumbers (*Cucumis sativus*) in Japan in 1975 and deposited at the Ministry of Agriculture, Fisheries and Forestry, Japan (MAFF no. 301315). The isolate used to derive strains for this report was directly acquired from MAFF, and the complete genome assembly of this strain was originally reported in Smith *et al.* [25] found at GenBank at accession GCA_000146005.2. Originally, a phenotypically marked version of strain *Pla*107 was created (named DAB885) through the integration of the vector pMTN1907 into a region on megaplasmid pMPPla107 and using positive selection for recombinants through tetracycline resistance [26,27]. Although pMTN1907 also contains an *aph*3A′ locus from *Campylobacter coli* with expression driven by its native promoter, and this locus enables kanamycin resistance in *Escherichia coli*, this gene as constituted does not enable *Pla*107 to grow on plates supplemented with kanamycin [28]. An isolate of this strain was grown overnight in King’s B medium (KB) supplemented with tetracycline (10 µg ml^−1^) on a shaking incubator at 27 °C at 220 r.p.m. After growth overnight, 200 µl of this culture was spread on KB agar plates supplemented with kanamycin (25 µg ml^−1^) and a single kanamycin-resistant colony was picked to KB liquid media. This culture was grown overnight and frozen down in the Baltrus Lab stock collection as strain DBL328. For genome sequencing, a subsample of this frozen stock was streaked to KB agar plates containing tetracycline and kanamycin, and subsequently a single colony was picked to KB liquid media supplemented with kanamycin. Genomic DNA from DBL328 was isolated from this liquid culture using a Promega (Madison, WI) Wizard kit, sequenced at Plasmidsaurus using standard protocols and assembled using their standard pipeline involving Flye v. 2.1 [29]. Sequencing reads used for this assembly have been deposited in the SRA (Sequence Read Archive) at accession SRR30528424. The genomic assembly of strain DBL328, generated from these reads, has been deposited at Figshare [1]. The coding sequence of the *aph*3A′ locus on the megaplasmid of pMPPla107 in DBL328 was queried by blastN in this assembly, with the region upstream of this locus analysed for promoter insertions.

### RNA isolation and RT-qPCR amplification

To analyse the expression of the kanamycin resistance locus in DAB885 and DBL328, three single colonies of each strain were individually picked to 2 ml KB media supplemented with tetracycline (10 µg ml^−1^), grown overnight at 27 °C with shaking and diluted 1:100 the next morning in KB media. Cultures were then grown at 27 °C with shaking for 5 h (until mid-log phase). Cells were spun for 3 min at 3,000 ***g***, the supernatant was removed and pellets were flash frozen in liquid nitrogen. Pellets were randomized and blinded for subsequent RNA isolation and RT-qPCR analysis. Pellets were resuspended by pipetting in 1 ml TRIzol (ThermoFisher Cat. No. 15596018) and held at room temperature for 5 min. Briefly, 200 µl chloroform was added, samples were shaken for 20 s, held at room temperature for 3 min, and then spun at 10,000 ***g*** for 18 min. 350 µl of the upper aqueous layer was transferred to a new tube, and 350 µl of 100% ethanol was added and mixed by pipetting. The mixture was then bound to an RNeasy column from a Qiagen RNeasy Mini kit (Cat. No. 74104) by centrifuging at 8,000 ***g*** for 30 s. The flow-through was discarded, and the sample was washed once with 700 µl Buffer RW1 from the RNeasy kit by centrifuging at 8,000 ***g*** for 30 s, then twice using 500 µl of Buffer RPE by centrifuging at 8,000 ***g*** for 30 s and 2 min, respectively, then eluted from the column in 40 µl ultrapure water. cDNA was synthesized from 1 µg of RNA using the ThermoFisher Maxima First Strand cDNA Synthesis Kit (Cat. No. K1671). cDNA was diluted 1:50 in nuclease-free water and used as template for RT-qPCR. A Bio-Rad CFX96 C1000 Touch thermocycler was used for RT-qPCR with the following programme: 95 °C 3 min, (95 °C 10 s, 60 °C 30 s)×40 cycles. RT-qPCR results were analysed using the Bio-Rad CFX Maestro software package. Statistical analysis and graphing were performed using GraphPad Prism. Primers used for kanamycin resistance locus RT-qPCR reaction were F: 5′-GGCTAAAATGAGAATATCACCGG-3′, R: 5′-CTTTAAAAAATCATACAGCTCGCG-3′ [30]. Primers used for tetracycline resistance RT-qPCR reaction were F: 5′-GCGGGATATCGTCCATTCCG-3′, R: 5′-GCGTAGAGGATCCACAGGACG-3′ [31].

### Statistics

Data for comparisons within Fig. 3(e) were analysed with a Student’s t-test, assuming equal variances. Data for comparisons within Fig. 3(f) were analysed with a one-way ANOVA, which produced an *F*=10.17, with groups ‘a’ and ‘b’ statistically significantly found to be different with a *P* value of 0.05 and a *P*=0.0042 by Tukey’s post-hoc test. Raw data for each analysis can be found at Figshare [1].

## Results

### Variability in presence and copy number of IS*5* elements throughout *P. syringae* complete genomes

**Fig. 1.**
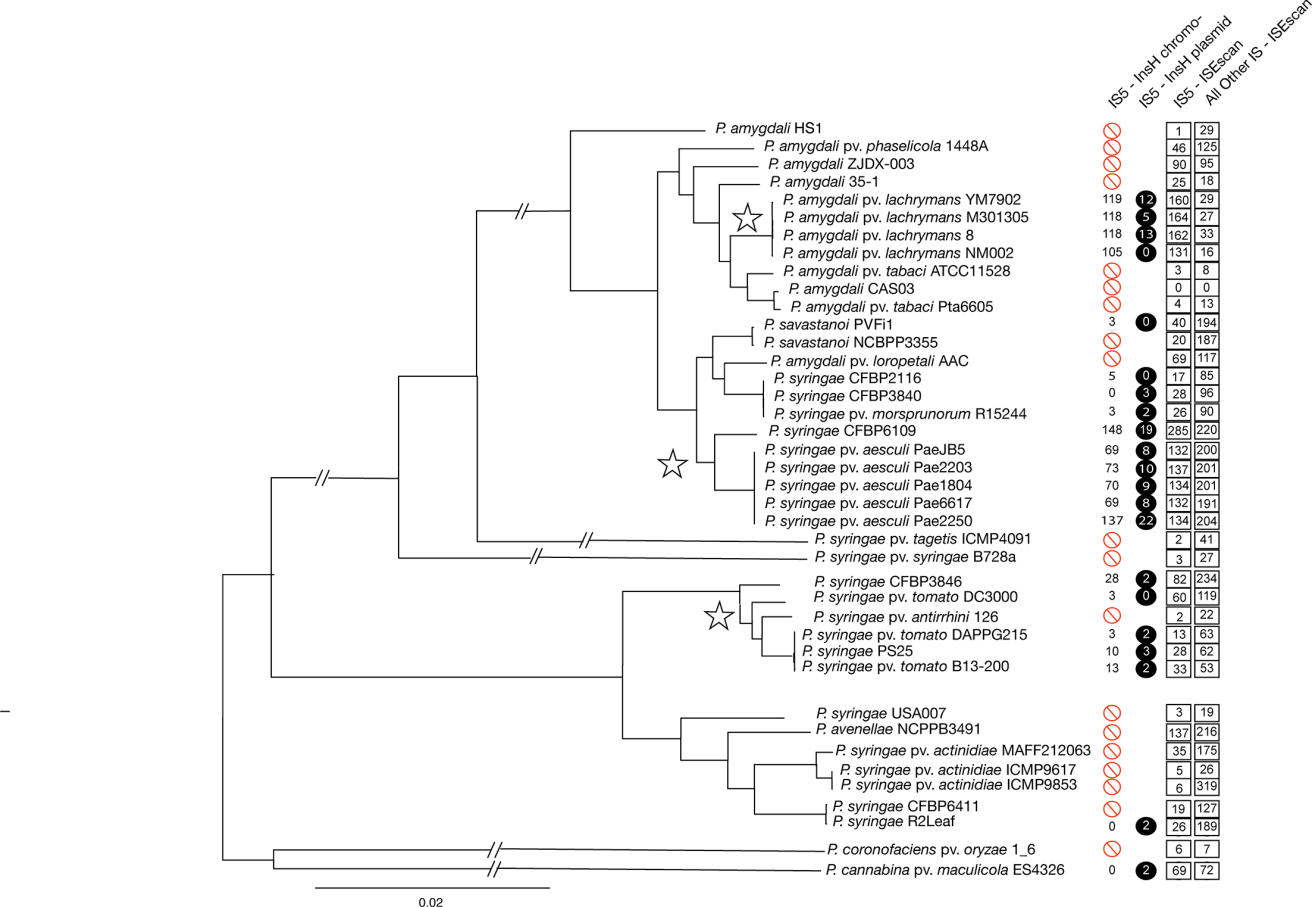
IS5 elements are found throughout *P. syringae* strains, with independent expansions across multiple clades. We used Realphy to infer a phylogeny across all complete *P. syringae* genomes containing the InsH transposase of the IS*5* element, while also including relevant representative complete genome sequences to adequately represent genomic diversity in this species. The first column displays either the number of IS*5* elements found on the chromosome of each strain (black numbers) or displays whether a genome lacks this element entirely (red circle). The second column (black circle with white number) displays the number of IS*5* elements that were localized to plasmids for most strains and to either plasmids or other fragments for *P. amygdali* pv. *aesculi* 2250. The third column displays the number of *all* IS*5* elements identified by ISEscan, while the fourth column displays the number of additional IS elements identified by ISEscan. Stars denote clades of interest for IS element expansions.

We used DiamondblastP to search all complete *P. syringae* (and related species) genomes through https://www.pseudomonas.com/ and to evaluate the presence and copy numbers of annotated InsH protein (the transposase of IS*5*, encoded within this IS element). Active IS*5* elements (with intact InsH) are not universally present throughout all *P. syringae* genomes, with their presence largely localized to three phylogenetic clades (with stars differentiating these clades highlighted in Fig. 1) in addition to a handful of other scattered strains. Two of these clades are found within phylogroup 3, while one clade is found within phylogroup 1. Copy numbers within each strain are quite variable, but we note that there has been a uniform explosion to >100 copies within two clades comprising two pathovars of *P. syringae* (pv. *aesculi*, a pathogen of horse chestnut; pv. *lachrymans*, a pathogen of cucurbits with these strains largely causative of disease in cucumber). Judging by phylogenetic relationships, and because of the absence of IS*5* elements within strains and clades separating *P. syringae* pv. *aesculi* and pv. *lachrymans*, a parsimonious explanation is that these IS elements independently expanded within each of these clades because there are numerous complete genome sequences that lack IS*5* elements interspersed between these two clades in the phylogeny.

We also note that IS*5* elements are often present on both the chromosome and plasmids of strains, although there are strains where the IS*5* element is present solely on the chromosome (*P. syringae* pv. *lachrymans* NM002, *Pseudomonas savastanoi*, *P. syringae* pv. *tomato* DC3000, *P. syringae* CFBP2116) as well as strains where this element is only present on the plasmid (*P. syringae* R2Leaf, *P. syringae* CFBP3840, *Pseudomonas cannabina* ES4326). Additionally, we acknowledge that listed numbers of *IS5* elements represent a minimum, as we counted only full-length and active (e.g. annotated) copies, and thus, pseudogenized versions will not be represented in this dataset. However, as an additional step, we searched genomes with no annotated IS*5* elements using tblastN with *Pla*107 InsH as a query and found zero cases where a genome only possessed pseudogenized versions of InsH (data not shown). If hits were present for InsH, they were weak (<50% sequenced identity) or internal fragments within annotated IS elements from alternative families. We therefore believe that the zero counts are an accurate representation of the number of these specific *Pla*107 IS*5* elements present within this dataset.

### Variability of IS element copy numbers throughout *P. syringae* complete genomes

IS element proliferation within a genome is hypothesized to potentially reflect trends in population biology for the lineages of interest [2,32, 33]. In this way, an explosion of IS*5* elements within a genome might reflect smaller effective population sizes for the lineages and clades highlighted by stars in Fig. 1. Since overall population dynamics should impact copy numbers of all IS element families (rather than just IS*5*), one clear prediction is that if IS*5* element expansion is driven by changes in population biology of the strains then all IS element families should be impacted and should therefore expand in concert within these genomes. To follow up on our initial investigation of the presence of intact *insH* (and IS*5* elements) across *P. syringae*, we further queried for the presence and copy numbers of all IS element families from complete genomes containing *insH* as well as additional phylogenetically informative strains. This query also allows independent enumeration of IS*5* elements, including those that are too divergent in InsH sequence to have been included in above analyses. Although InsH protein sequences from IS*5* elements used in queries above appear to be nearly identical in sequence within and across all genomes where present, with only one or two amino acid substitutions differentiating alleles of *insH*, it is likely that there are a variety of distinct and highly diverged IS*5* elements also found within some genomes. Judging by distinct allelic classes of these IS*5* elements, it is highly likely that divergent copies have arisen from a separate horizontal gene transfer event into these strain backgrounds. From the data, there is no clear signal of correlation between IS*5* copy number and copy numbers of other IS element families within each of the lineages. Strains in *P. syringae* pv. *lachrymans* with large numbers of IS*5* elements contain a relatively small number of additional IS element families. Conversely, strains in *P. syringae* pv. *aesculi* contain large numbers of both IS*5* elements and other additional IS elements. Strains in phylogroup 1 with subtle increases in IS*5* copy numbers (between 13 and 30) can contain relatively few (53) to many (234) additional IS element copies. Thus, we do not find a clear general signal that can explain IS*5* expansion. This hints that lineage level complexities in population and genome dynamics could be the main drivers of proliferation of IS elements across strains.

### Specific proliferation and movement of IS*5* elements in three *P. syringae* pv. *lachrymans* genomes

To evaluate the variability in the position of IS*5* elements across closely related strains, we compared IS*5* element distributions from the subclass queried above (represented by InsH sequences) of three *P. syringae* pv. *lachrymans* chromosomes. These three strains are closely related according to phylogenetic relationships (Fig. 1) and display Average Nucleotide Identity (ANI) values >99% in all pairwise comparisons (see heatmap in Fig. S1, calculated by FastANI at GTDB [34,35]). Although we sought to classify conservation of each IS*5* element according to synteny across genomes, genomic variability in regions containing IS*5* elements across strains made many of these comparisons challenging. Thus, we split IS*5* elements into classes based on distributions across all three genomes and further split each locus within each class into groups (within the presence/absence split) depending on whether synteny was ‘clear’ or ‘unclear’.

**Fig. 2.**
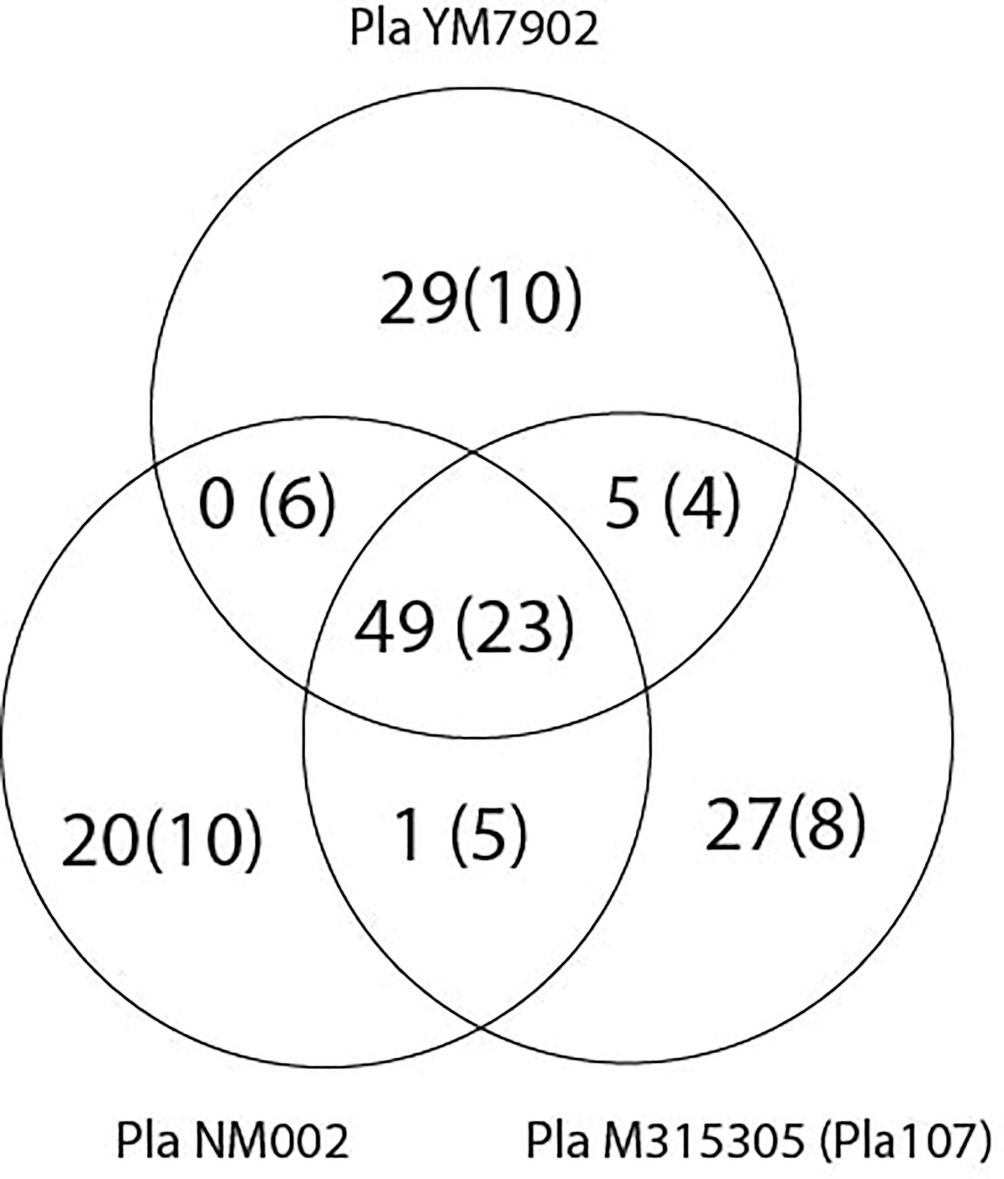
Diversity in IS*5* element insertion sites across three *P*. *amygdali* pv. *lachrymans*. We identified whether IS*5* element insertion points (represented by annotated InsH protein sequences) were conserved or divergent across the chromosomes of three strains within pv. *lachrymans*: M315305 (*Pla*107), NM002, YM7902. The number outside of parentheses indicates situations where IS*5* elements are shared or divergent between strains with high confidence (the clear annotation group), while the number inside parentheses indicates the number of lower confidence instances (the unclear annotation group).

As shown in Fig. 2, we found that 49 of the IS elements are found in the same positions across each of the 3 genomes, while 23 additional loci appear to be potentially conserved in position across the 3 strains, but where syntenic relationships are unclear. Given that there are >100 IS*5* elements within each strain, a minority of all IS*5* elements in each genome are present in conserved locations across these three strains. Further, each strain possessed between 20 and 30 copies of the IS*5* element which were present in unique locations across their genomes with an additional ~10 copies per strain where it was likely that the loci were uniquely present. Many of the unique insertions were in regions of the chromosome that were variable mobile elements (predicted to be integrated conjugative elements and phage regions), while numerous others were the product of local tandem duplications of a conserved IS*5* element (data not shown).

### IS*5* elements within *Pla*107 are capable of driving downstream gene expression

**Fig. 3.**
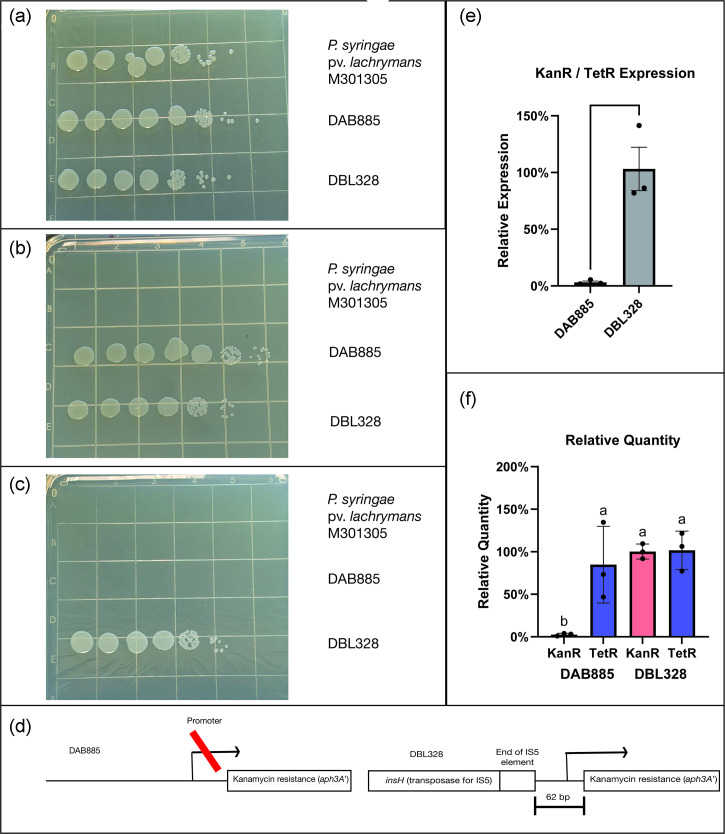
IS*5* elements can drive downstream gene expression. Strain DAB885 is an isolate of *P. amygdali* pv. *lachrymans* M301305, in which pMTN1907 has been recombined into a megaplasmid found in the strain. pMTN1907 codes for an enzyme that provides kanamycin resistance, but which is not expressed in *P. syringae*. We selected for a kanamycin-resistant version of DAB885 by plating cells onto KB media containing 25 µg ml^−1^ kanamycin to generate strain DBL328. (a)–(c) We show growth after dilution plating overnight cultures of either *P. amygdali* pv. *lachrymans* M3013015, DAB885 and DBL328 onto KB media, KB media containing 10 µg ml^−1^ tetracycline or KB media containing 25 µg ml^−1^ kanamycin. (d) Genome sequencing demonstrated that an IS*5* element transposed upstream of the kanamycin resistance gene within DBL328 to enable kanamycin resistance. (e) Relative expression of the *aph*3A′ locus compared to the *tetA* locus in strains DAB885 and DBL328. A Student’s t-test was used for statistical comparisons across three biological replicates. (f) Relative quantity of mRNA for *aph*3A′ (KanR) and *tetA* (TetR) in strains DAB885 and DBL328 at mid-log phase. Data from three biological replicates are shown. Different letters indicate statistical differences between groups by XXXX.

IS*5* elements have previously been shown to be able to drive gene of downstream genes in other bacterial species. We therefore took advantage of a previously constructed strain to act as a promoter trap to select this ability in IS*5* elements from *P. syringae*. The genome of *Pla*107 contains a mobilizable megaplasmid that we have previously tagged using the suicide vector pMTN1907. pMTN1907 contains ORFs capable of encoding resistance to both kanamycin (*aph*3A′) and tetracycline (*tetA*), and replication of this plasmid within *E. coli* provides resistance to both of these antibiotics. However, whereas the tetracycline resistance locus is expressed and provides resistance when this vector is incorporated into the genome of *Pseudomonas* strains, the *aph*3A′ gene is not expressed in *Pseudomonas* and therefore strains with pMTN1907 recombined into their chromosomes (or other replicons) remain kanamycin sensitive. We hypothesized that differential functions of the promoter for kanamycin resistance within this plasmid could explain differential resistance phenotypes in *E. coli* and *Pseudomonas* strains. Therefore, we selected for kanamycin-resistant mutants of our previously constructed *Pla*107::pMTN1907 strain (DAB885) with the hopes that we could identify a transposition event of IS*5* elements in kanamycin-resistant isolates of this strain. We found that kanamycin resistant mutants occurred quite frequently within this strain background, and sequencing and assembly of one of these revertants demonstrated insertion of an IS*5* element upstream of the kanamycin resistance gene within the version of pMTN907 found on the megaplasmid. Specifically, it appears as though a 1,210 bp IS*5* family element transposed into a position 62 bp upstream of the start codon for kanamycin resistance in the integrated version of pMTN1907 (Fig. 3a).

To confirm our prediction that the IS*5* element did indeed drive expression in strain DBL328, we performed RT-qPCR using primers anchored in both the *tetA* locus and *aph*3A′ in both the previously constructed *Pla*107::pMTN1907 strain (DAB885) as well as the kanamycin-resistant derivative DBL328. Our data support the hypothesis that the IS*5* element can drive gene expression of *aph*3A′ in this context since the *tetA* locus is equally upregulated in both backgrounds in mid-log phase, but *aph*3A′ is only expressed in strain DBL328 (Fig. 3e and f).

## Discussion

### Ecological and population-level inferences of IS element proliferation

We present data demonstrating that IS*5* elements are undergoing ongoing and independent expansion across multiple phylogenetic clades of *P. syringae*. What is less clear are the evolutionary and ecological forces that enable such expansions within these specific clades. One possibility is that IS*5* elements have not expanded within other clades simply because these elements have been introduced to genomes relatively recently and there therefore hasn’t been enough time for expansions to occur. As an argument against this relatively simple explanation, we highlight that clades which have undergone expansion (highlighted by stars in Fig. 1) contain equal or less diversity across strains (are similarly aged or ‘younger’) than another clade with more moderate IS*5* element copy numbers as judged by phylogenetic distance.

It is also possible that there are particularities about the ecology of pv. *lachrymans* and pv. *aesculi* that enable expansion of IS elements compared to other clades. Although IS element insertions can be beneficial under certain contexts and circumstances [3,16, 36], it is thought that these events occur in a minority of cases and that the IS element copy number within a genome is driven by a combination of neutral and deleterious transposition events [33,37, 38]. At one end of the spectrum, transposon landing sites are restricted to regions of the genome that are not critical for carrying out cellular functions within a given environment because disruption of these regions is lethal. However, if a transposition event is non-lethal to the cell but either partially lowers fitness of the cell or is neutral, the strength of selection against this particular insertion site at a population level will be driven by population size [39]. At relatively large effective population sizes, selection is a powerful force to cull most deleterious mutations from a population, and genetic drift is comparatively weak, thus limiting the fixation of neutral mutations. However, at relatively small effective population sizes, selection is much weaker and genetic drift much stronger, and thus deleterious and neutral mutations can fix more easily [40]. Thus, it may be that pv. *lachrymans* strains and pv. *aesculi* clades possess smaller effective population sizes than other clades due to ecological differences of these strains. Perhaps they undergo smaller or more frequent population bottlenecks during transmission and infection.

To this point, we highlight that both pv. *lachrymans* and pv. *aesculi* clades are part of phylogroup 3 of *P. syringae*. Although many *P. syringae* strains have been isolated from environmental and water sources, and it is often assumed that their life cycles are intimately tied to transmission by the water cycle, it is notable that relatively few phylogroup 3 strains have been isolated from water sources compared to other phylogroups [19,41]. Thus, phylogroup 3 strains may have distinct transmission mechanisms which could have cascading effects on their population dynamics and drive smaller effective population sizes and enable IS5 element proliferation. While likely affected by sampling bias, most of the dramatic cases of IS element and transposon expansions in bacterial genomes thus far are found in obligate symbionts and parasites whose population sizes are drastically bottlenecked during transmission from host to host [40,42]. Flipping this idea around, differential IS element proliferation in one bacterial lineage compared to a closely related lineage could therefore be an indicator of a change of relevant evolutionary parameters in populations from one lineage compared to the other. However, we note that our data also suggest that collections of IS element families are not impacted equally across each lineage. Therefore, if changes in population dynamics have enabled expansions of IS*5* elements in both pvs. *lachrymans* and *aesculi*, the population-level phenomena enabling such expansions have not affected all IS element families similarly, as broad IS element expansion has only occurred in pv. *aesculi* strains. While our data suggest that strains within pv. *aesculi* may display different population dynamics than other lineages of *P. syringae* as reflected in IS element copy numbers, changes in population parameters therefore do not cleanly and generally explain expansion of IS*5* elements for pv. *lachrymans*.

To our knowledge, there have been few previous attempts to characterize IS element proliferation across multiple closely related genomes at both the copy numbers and granularity of genome context reported in this manuscript. Previous reports have typically focused on comparing overall numbers of IS elements between genomes of various distances [23], have identified shared and divergent locations of IS elements across genomes with lower transposon numbers overall [43] or have done so in a manner that emphasizes identification of new insertions [44]. Our results, that IS element positions can differ dramatically across highly related genomes, echo narratives arising from these reports. Moreover, our data highlight that IS elements can rapidly proliferate from a very small (<5) to much higher (>100) copy numbers over relatively short periods of time even within closely related bacterial species and that positions of many of these insertions can dramatically differ between strains.

We acknowledge that our reported results on IS*5* element lineage-specific expansion could be explained in part through the existence of additional mechanistic phenomena acting in a highly specific manner. A recent manuscript suggested that IS*5* elements could undergo a ‘copy/paste’ mechanism of transposition within a genome rather than the zero-sum ‘cut/paste’ mechanism [45]. Through this action, a single copy of an IS*5* element may be able to copy itself and jump to a different genomic location in addition to being maintained at the original insertion site, especially if present on a multi-copy replicon. Such a replication mechanism could facilitate rapid and accelerated expansion of these elements over relatively short evolutionary periods, but it is unclear why this effect would be lineage-specific if IS*5* elements are already present. Alternatively, it is possible that there is interference of IS*5* elements by other proteins or IS elements in a lineage-specific manner, with expansions therefore explained by absence of inhibition in certain strains or lineages. It is well established that enzymes exist which can limit transposon activity [46] and that chromosome structure and/or protein activity can bias transposition [47]. However, there is limited data supporting this hypothesis at present for this system as there are no known enzymes that directly limit *IS5* transposon activity [17]. In the absence of additional future experiments this explanation remains intriguing but unfounded.

### Influence of IS element proliferation on evolutionary dynamics

Aside from possible ecological correlates of IS*5* element proliferation, what are the possible evolutionary consequences of such expansions? When IS elements proliferate throughout bacterial genomes, they seed that genome with many copies of identical ~1,000 bp nucleotide sequences. These sequences can then act as landing sites for homologous recombination events, including duplications, deletions and inversions [2,48, 49]. Therefore, regardless of changes in population dynamics that affect the fixation of mutations, *P. syringae* lineages where specific IS element families have proliferated to hundreds of copies could therefore be prone to a higher frequency of occurrence of these types of events through time and could be considered to have higher evolutionary plasticity than sister lineages without high levels of IS element proliferation. Indeed, it is worth mentioning that one of the difficulties in identifying synteny between IS*5* element insertions is that the regions of the genome they are found in are prone to duplications, inversions and translocations.

Additionally, one of the most intensely investigated aspects of IS*5* element biology is the ability of this family of elements to enable the expression of downstream genes and operons, and this has largely been described as occurring in multiple operons in *E. coli* strains [16,18, 36]. To our knowledge, our data represent one of the clearest demonstrations that IS*5* elements can lead to upregulation in systems outside of the well-studied *E. coli* models and could provide useful evolutionary comparison for dissecting the upregulation phenomenon at a systems level. It is currently unknown how many of the ~>100 copy numbers of IS*5* elements actually do drive downstream gene expression in *P. syringae* pv. *lachrymans* and *aesculi* genomes, but we expect future studies to better characterize the potential of these elements to change gene expression patterns.

**Fig. 4.**
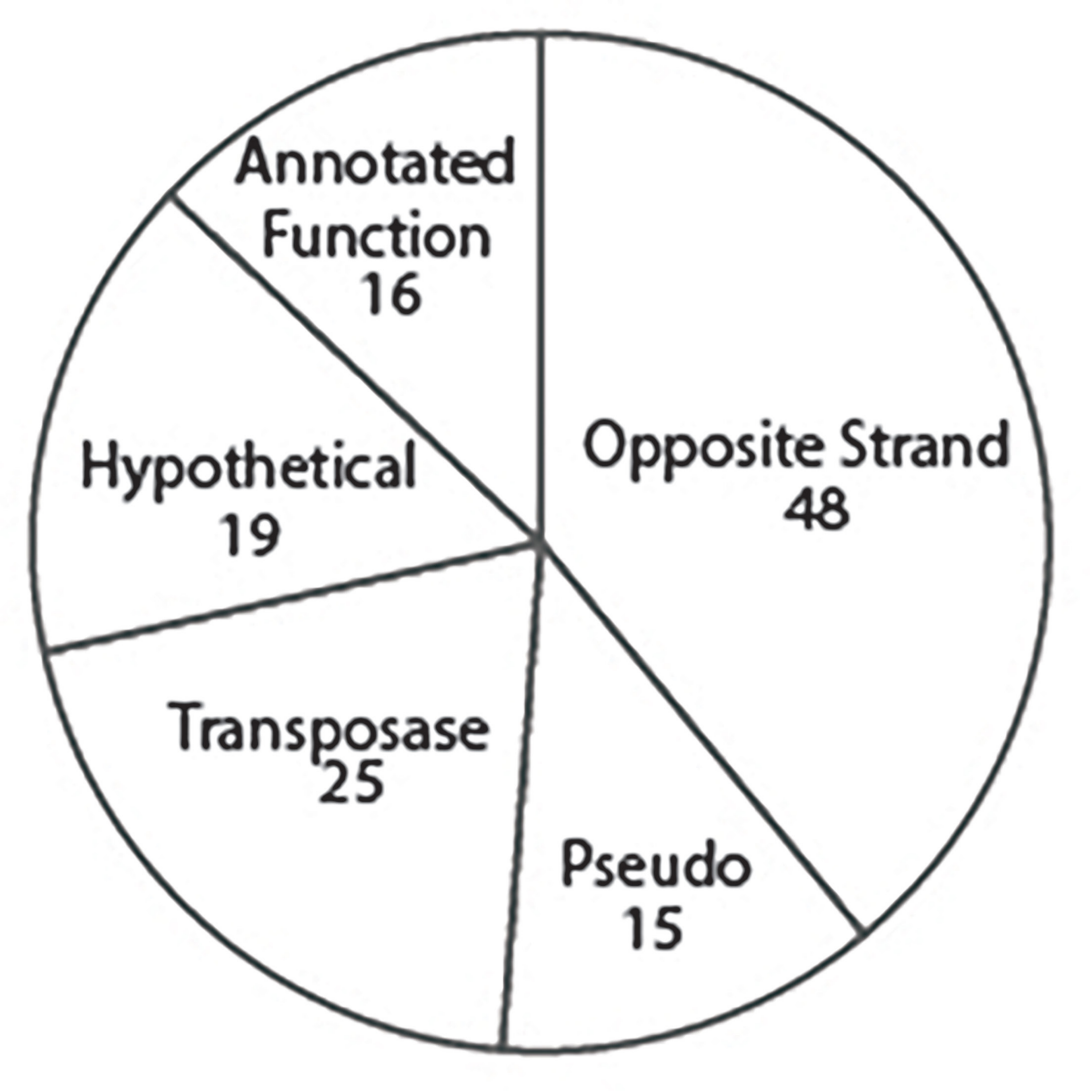
Characterization of IS*5* element insertion sites and downstream genes. We have characterized genes downstream of all IS*5* element insertions in the genome of *P. syringae* pv. *lachrymans* 107. The ‘Wrong context’ category represents situations where the IS*5* element is on the opposite DNA strand as a potential proximate downstream gene. The ‘Transposase’ category represents situations where the IS*5* element is immediately upstream of another potential IS element on the same strand. The ‘Hypothetical’ category represents situations where there is a potential gene downstream of the IS*5* element, but this gene is annotated as hypothetical. The ‘Annotated Function’ category represents genes which are downstream of the IS*5* element and are on the same strand of DNA and which have annotated functions other than transposase activity. The ‘Pseudo’ category indicates situations where IS*5* elements are either upstream of annotated pseudogenes or have clearly disrupted a previously intact locus (upstream and downstream genes of the IS*5* element have the same annotation).

We have further investigated potential influences of IS*5* insertions within the *P. syringae* pv. *lachrymans* 107 by categorizing genes immediately downstream of all IS*5* insertions in this genome. Our assumption in this analysis is that the IS*5* element must be positioned upstream of the gene and on the same strand to enable upregulation (the same position as in DBL328 upstream of *aph*3A′). We acknowledge that this assumption could be wrong and that alternative positioning of IS*5* elements could lead to upregulation of proximate genes, but we feel as though such speculation is out of scope for this current manuscript. As one can see in Fig. 4, a majority of the IS*5* insertions are located on the opposite strand of DNA as the next potential downstream gene and thus are not currently candidates for upregulation by these elements if our assumption about position holds. The second largest group of genes downstream of IS*5* elements is additional copies of IS elements (with both IS5 and IS*Psy*19 represented). Given the large number of IS elements clustered in this manner, we believe it is likely that these elements may be jumping and proliferating throughout these genomes as compound transposons or at least in a correlated manner. The third largest class of genes downstream of IS*5* elements is annotated as ‘hypothetical’ proteins, while the fourth class likely represents genes with a high likelihood of coding for proteins. Although this fourth class is relatively small, we note that it does appear to be overrepresented in DNA-binding elements and those involved in metabolism (Table 1), and it will be interesting to investigate IS element influences on their expression patterns across strains.

**Table 1.** Annotated loci downstream of IS*5* elements in *P. syringae* pv. *lachrymans* 107

Locus tag of IS*5* element	RefSeq ID of downstream gene	Distance between InsH and gene (bp)	Functional annotation
PLA107_RS09245	WP_005780873.1	285	GO:0006935 – chemotaxis
PLA107_RS09315	WP_005745291.1	184	GO:0003700 – DNA-binding transcription
PLA107_RS10475	WP_005745577.1	513	GO:0008168 – methyltransferase activity
PLA107_RS11585	WP_005743835.1	260	GO:0016740 – transferase activity
PLA107_RS15180	WP_005745213.1	264	GO:0004222 – metalloendopeptidase activity
PLA107_RS18200	WP_002553945.1	228	Alpha/beta fold hydrolase MenH
PLA107_RS20965	WP_005744171.1	802	GO:0016746 – acyltransferase activity
PLA107_RS23725	WP_005743264.1	254	DABB family protein
PLA107_RS24535	WP_002554444.1	328	GO:0003987 – acetate-CoA ligase activity
PLA107_RS25360	WP_002554584.1	413	GO:0016432 – tRNA-uridine aminocarboxypropyltransferase activity
PLA107_RS25860	WP_004657623.1	154	GO:0003677 – DNA binding
PLA107_RS27250	WP_002552171.1	320	GO:0009116 – nucleoside metabolic process
PLA107_RS28260	WP_101165381.1	264	GO:0008754 – O antigen ligase activity
PLA107_RS28390	WP_005745459.1	850	GO:0003677 – DNA binding
PLA107_RS28465	WP_005745444.1	272	GO:0022857 – transmembrane transporter
PLA107_RS32845	WP_005746935.1	290	3-oxoacyl-[acyl-carrier-protein] synthase III

## Conclusions

Increasing numbers of complete bacterial genomes provide an exceptional opportunity to take account of IS element variation across strains and within bacterial species and here we evaluate the distribution of IS*5* elements across the phytopathogen *P. syringae*. Our report demonstrates that IS*5* elements are prevalent within the genomes of a variety of lineages of *P. syringae*, that some lineages display independent expansions of this element, that these elements are actively transposing throughout these genomes such that a minority of insertion sites are conserved across closely related strains and that this element has the potential to drive downstream gene expression within these strains. Moreover, many of the insertion sites that are unique to specific strains are present within regions of the genome which are differentially present or absent from closely related genomes, and regions of IS*5* insertions are often sites that often show inversions and duplications/deletions. At present, it is unclear whether changes in population-level dynamics can explain the differential expansion of IS*5* elements within pvs. *aesculi* and *lachrymans* strains. It also remains unknown what fraction of the ~100 copies of IS*5* elements within some genomes directly upregulate expression within downstream regions.

## Abbreviations

IS: insertion sequence
KB: King’s B medium
MAFF: Ministry of Agriculture, Fisheries and Forestry
